# Nanoscale *Operando* Imaging of Electrically Driven Charge-Density Wave Phase Transitions

**DOI:** 10.1021/acs.nanolett.4c03324

**Published:** 2024-09-24

**Authors:** Till Domröse, Noelia Fernandez, Christian Eckel, Kai Rossnagel, R. Thomas Weitz, Claus Ropers

**Affiliations:** † Department of Ultrafast Dynamics, Max Planck Institute for Multidisciplinary Sciences, 37077 Göttingen, Germany; ‡ 4th Physical Institute − Solids and Nanostructures, University of Göttingen, 37077 Göttingen, Germany; ¶ 1st Institute of Physics, University of Göttingen, 37077 Göttingen, Germany; § Institute of Experimental and Applied Physics, Kiel University, 24098 Kiel, Germany; ∥ Ruprecht Haensel Laboratory, Deutsches Elektronen-Synchrotron DESY, 22607 Hamburg, Germany; ⊥ International Center for Advanced Studies of Energy Conversion (ICASEC), University of Göttingen, 37077 Göttingen, Germany

**Keywords:** structural phase transformations, strongly correlated materials, charge-density waves, transmission electron microscopy, nanoscale *operando* imaging, electrically induced phase transitions

## Abstract

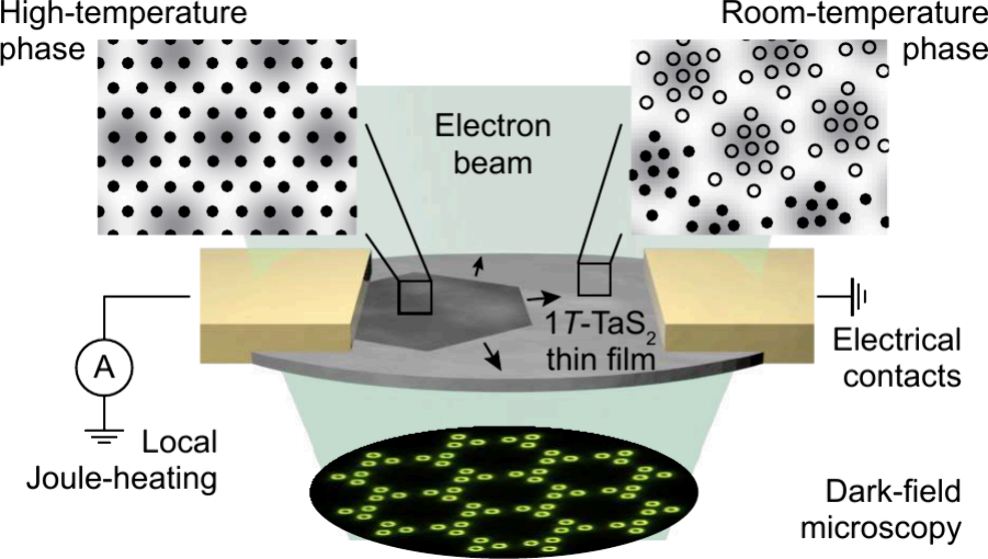

Structural transformations in strongly correlated materials promise efficient and fast control of materials’ properties via electrical or optical stimulation. The desired functionality of devices operating based on phase transitions, however, will also be influenced by nanoscale heterogeneity. Experimentally characterizing the relationship between microstructure and phase switching remains challenging, as nanometer resolution and high sensitivity to subtle structural modifications are required. Here, we demonstrate nanoimaging of a current-induced phase transformation in the charge-density wave (CDW) material 1*T*-TaS_2_. Combining electrical characterizations with tailored contrast enhancement, we correlate macroscopic resistance changes with the nanoscale nucleation and growth of CDW phase domains. In particular, we locally determine the transformation barrier in the presence of dislocations and strain, underlining their non-negligible impact on future functional devices. Thereby, our results demonstrate the merit of tailored contrast enhancement and beam shaping for advanced *operando* microscopy of quantum materials and devices.

Macroscopic materials’ properties are intrinsically tied to the underlying atomic arrangement. A structural phase transformation connecting different states via the tuning of an external parameter thus offers exciting prospects for technological applications.^1,2^ The development of functional devices, however, requires an understanding of how the microstructure affects phase switching as, for example, phase boundaries interact with defect sites or interfaces.^3−6^ At the same time, nanoscale heterogeneity may also foster functionality, exploiting phase coexistence^7^ or catalytically active surfaces.^8−10^ Understanding the fundamental working principles behind these processes necessitates experimental tools distinguishing the phases involved and resolving the structural transformations on their intrinsic time and length scales.^11−16^

As a prominent example, data storage in phase-change materials is based on the thermally induced transformation and associated distinct resistance difference between amorphous and crystalline order.^17−20^ Temporally, the sub-nanosecond write- and read-out speed is limited by the crystal domain nucleation and growth.^21−23^ The length scales involved are accessible by transmission electron microscopes (TEMs). Beyond atomic resolution approaches,^18^ nanoscale imaging of the transitions relies on the vastly different diffraction contrast associated with the two phases.^24,25^

Transformations between structurally similar phases promise even faster switching times and lower energy consumption.^26,27^ In charge-density wave (CDW) systems, the modulation of the crystal charge density is coupled to a periodic lattice distortion (PLD).^28^ The formation of these superperiodicities often results in transitions from metallic to semimetallic or insulating behavior, connected to sub-angstrom atomic displacements.^29^ The prototypical material 1*T*-TaS_2_, for example, features a rich phase diagram including several CDW phases, indicative of a competition between various coupled charge and lattice orders that is tunable by external control parameters.^30−32^ Structural transformations between these phases unfold as fast as a few hundred femtoseconds,^33,34^ and ultrashort optical or electrical pulses can bring about thermally inaccessible, metastable states.^34−39^

Transitions between different PLDs can also be induced electrically,^31,40−56^ foreshadowing possible applications^47−49,57−60^ that complement devices employing the strong correlations between electrons, phonons, and spins in related compounds.^8,61−63^ Due to the relatively low and often similar energy scales involved, nanoscale heterogeneity may critically affect future device performance. Specifically, CDWs are highly susceptible to strain^64,65^ that not only can induce transformations between competing phases^66,67^ but may also govern the phase nucleation^68^ by lowering both critical temperatures and energy barriers.^6,69^ Accordingly, local variations in switching, e.g., in the vicinity of dislocations, are to be expected. In the past, experimental characterizations of CDW transformations involved, on the one hand, spatially averaged observables in, for example, spectroscopy^6^ or elastoresistivity measurements.^66^ On the other hand, surface-sensitive investigations of the phase switching on micro- to nanometer lengths scales^46,51,70^ revealed a correlation between the transition temperature and spatial heterogeneity,^71^ calling for direct experimental access to the material’s microstructure during the current-induced transition. Fewer investigations combine such structural contrast with nanoscale access to the PLD amplitude,^72^ finding pinning of incommensurate CDWs at dislocations after a completed phase switch^54^ and mesoscale structural dynamics induced by sample heating upon biasing.^55^ Observing the electrically induced transformation *in situ*, allowing to characterize the influence of strain on the phase nucleation and the interaction of the propagating phase front with defects, however, remains an open challenge. In particular, such experiments require an increase in the experimental sensitivity to the small structural contrast variations associated with the atomic reconfigurations.

In this work, we image a CDW phase transformation in two electrically contacted 1*T*-TaS_2_ thin films *in-operando* by means of selective contrast enhancement tailored to nanoscale modifications of the PLD amplitude. Combined electrical transport measurements and TEM imaging of the current-induced phase switching allows us to link macroscopic resistance changes to the nucleation and subsequent growth of the high-temperature phase domain. We quantify local hysteresis to obtain the spatial variation of the phase transformation barrier with 5 nm spatial resolution. Current-induced phase nucleation is shown to be enhanced in regions of elevated local strain and a high density of basal dislocations. To provide a strategy for controlled switching without spontaneous nucleation, we present a second device geometry featuring deterministic thermal seeding and global hysteresis. Our results demonstrate the importance of selective contrast enhancement for the characterization of functional devices and materials in *operando* or *in situ* electron microscopy.

In our measurements, selective CDW phase contrast is established by an analog filter, a well-established approach in transmission electron microscopes. Specifically, such dark-field (DF) imaging exploits the spatial separation of diffracted beams in the back-focal plane of the objective lens (Figure 1a).^73−76^ Typically, the employed DF masks for trimming the electron signal are rotationally symmetric, in the form of either an individual circular aperture^73,74^ or a central beam stop.^75,76^ Aligning the DF filter to the electron diffractogram then allows for real-space imaging based on the selected electron momentum changes induced by the specimen. Similar optical elements have also been employed in DF momentum microscopy,^77^ second-harmonic generation,^78^ X-ray imaging,^79^ and neutron tomography.^80^

**Figure 1:**
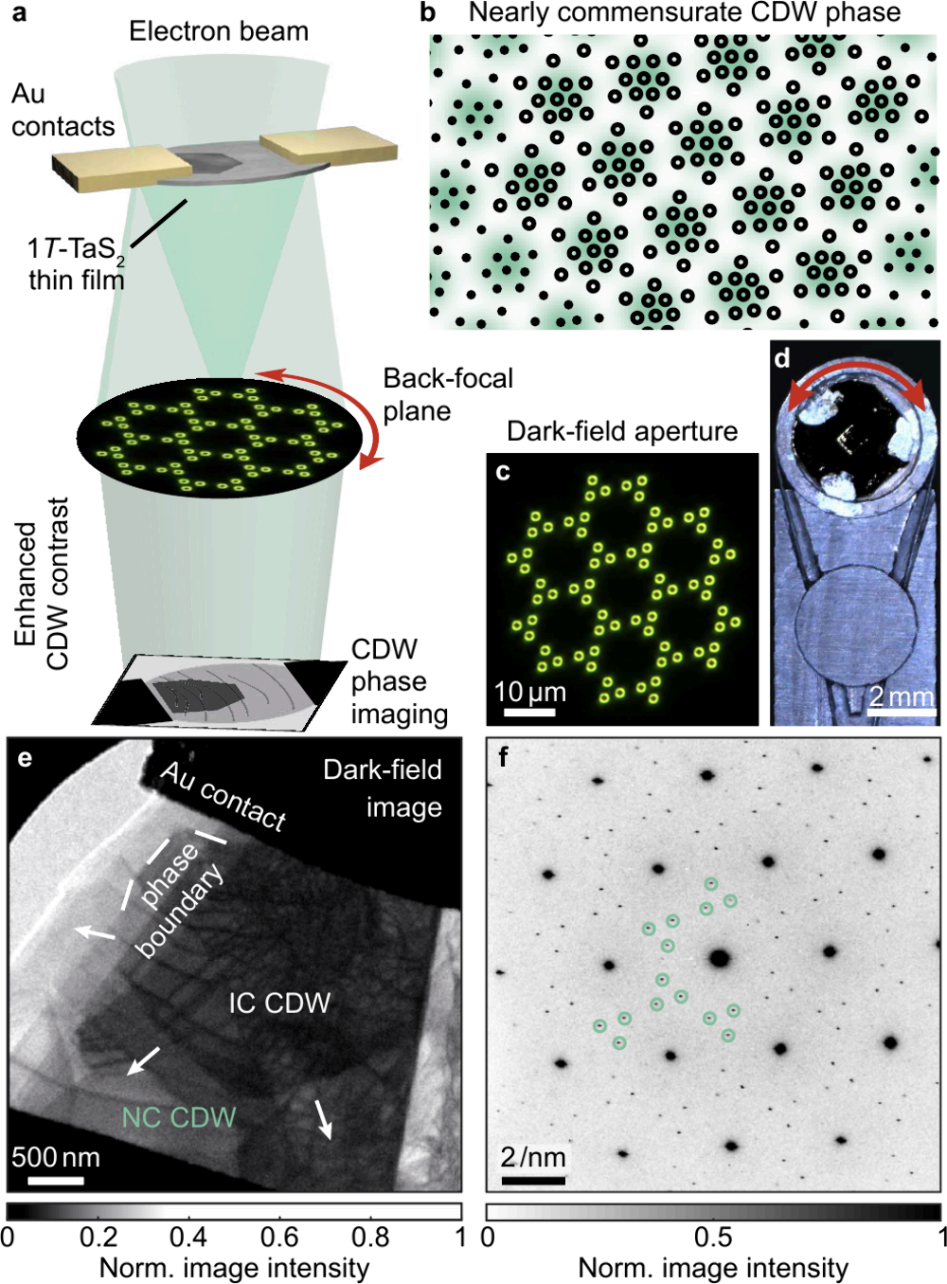
Dark-field imaging of charge-density wave phase transitions. **a** Measurement scheme. Contrast enhancement in the back-focal plane of the objective lens via a tailored dark-field (DF) aperture resolves a structural transformation in an electrically contacted 1*T*-TaS_2_ thin film. **b** Nearly commensurate (NC) phase of 1*T*-TaS_2_. The charge-density modulation (colored background) is coupled to a periodic lattice distortion (PLD, exaggerated five times; black symbols: tantalum sublattice). Commensurately distorted regions (empty circles) are separated by discommensurations (closed circles). **c** Microscopy image of the DF aperture. Only electrons scattered by the NC periodicities pass the analog filter. **d** A custom aperture holder allows the DF mask to be aligned with the electron diffractogram. **e** DF image for an applied bias of 908 mV. The structural transition locally reduces the image intensity. **f** Electron diffractogram in the NC phase. DF-filtering for the signature low-intensity satellites (green circles) yields direct CDW phase information and nanometer resolution of the PLD amplitude.

Phase transitions, however, usually involve structural changes that affect the entire *k*-space representation of the electron beam, particularly those between different crystalline states. More complex filtering schemes thus bear the potential for further contrast enhancement, which is especially beneficial for imaging transitions encoded in low-intensity signatures. PLD superstructures, for example, can be identified in electron diffractograms by additional reflections around every bright diffraction spot in the undistorted host lattice (Figure 1f). DF imaging based on these satellites yields a local measure of the PLD amplitude, but sufficient image contrast only arises from the contribution of numerous diffracted beams to the detected signal due to the small PLD structure factor.^81^

Recently, we introduced such a tailored imaging scheme in an investigation of laser-induced phase switching in 1*T*-TaS_2_.^82^ At room temperature, the material’s nearly commensurate (NC) CDW phase is characterized by prominent higher harmonics of the modulation wave vector that bring about a domain-like phase pattern (Figure 1b). Regions with commensurate lattice distortions, closely resembling the low-temperature phase of the material, are separated by a network of discommensurations.^83^ In bulk samples, the NC CDW additionally forms a 3-fold stacking sequence.

An example diffractogram of the NC phase taken along the [001] zone-axis is depicted in Figure 1f. Second-order satellites (highlighted by green circles) are the most prominent PLD features under this illumination, while first-order spots are situated in higher-order Laue zones.^83^ The transformation into the high-temperature incommensurate (IC) phase above 352 K results in a rotation of the PLD wavevector by around 12°^29^ such that the two phases can be distinguished by the position of the PLD spots in reciprocal space. Our tailored DF imaging approach is based on filtering the diffracted signal with an array of 72 individual apertures whose distribution corresponds to the position of the brightest NC reflections in the diffractograms^82^ (Figure 1c). The DF filter alignment is enabled by a custom-made aperture holder that allows for a 360° in-plane rotation (Figure 1d; see also Supporting Information). Thus, our measurement scheme is also applicable to other forms of *in situ*(4,5,11,12,14,84−89) or *operando* investigations.^9,10,90^

In the following, we make use of these experimental possibilities and directly image the NC-to-IC phase transformation, characterizing its interaction with the host material’s microstructure. The investigated sample consists of a single-crystal 1*T*-TaS_2_ thin film that is electrically contacted by two gold leads. An example DF image with an applied bias of 908 mV is depicted in Figure 1e. The 1*T*-TaS_2_ flake shows a spatially varying density of basal dislocations, apparent from image intensity deficiency lines. The visibility of these features is increased after excluding the high-angle diffuse scattering from the image formation by means of the analog filter.^73^ The spatial heterogeneity in the device thus extends over a range of length scales, from few-nanometer dislocation distances to tens-of-nanometer-wide strain profiles and dislocation lengths, up to the micrometer separation between the gold contacts. Most importantly, the structural transformation is recognizable from a pronounced intensity suppression by ∼30% in the switched region. Furthermore, the interphase boundary is resolved with 5 nm precision,^82^ allowing to correlate the different degrees of heterogeneity to the structural transformation.

Figure 2 displays representative frames from a measurement where we slowly increase the applied voltage from 700 mV to a maximum of 960 mV and back (see Supplementary Videos 1 and 2 and Figure S5 for voltage-dependent electron diffractograms). Difference images obtained by subtracting a reference frame taken before IC phase nucleation (Figure 2f–i) further illustrate the DF contrast. With voltage steps of 2 mV during the up- and the down-sweep followed by an acquisition time of 60 s, the system is captured in a stationary state.

**Figure 2:**
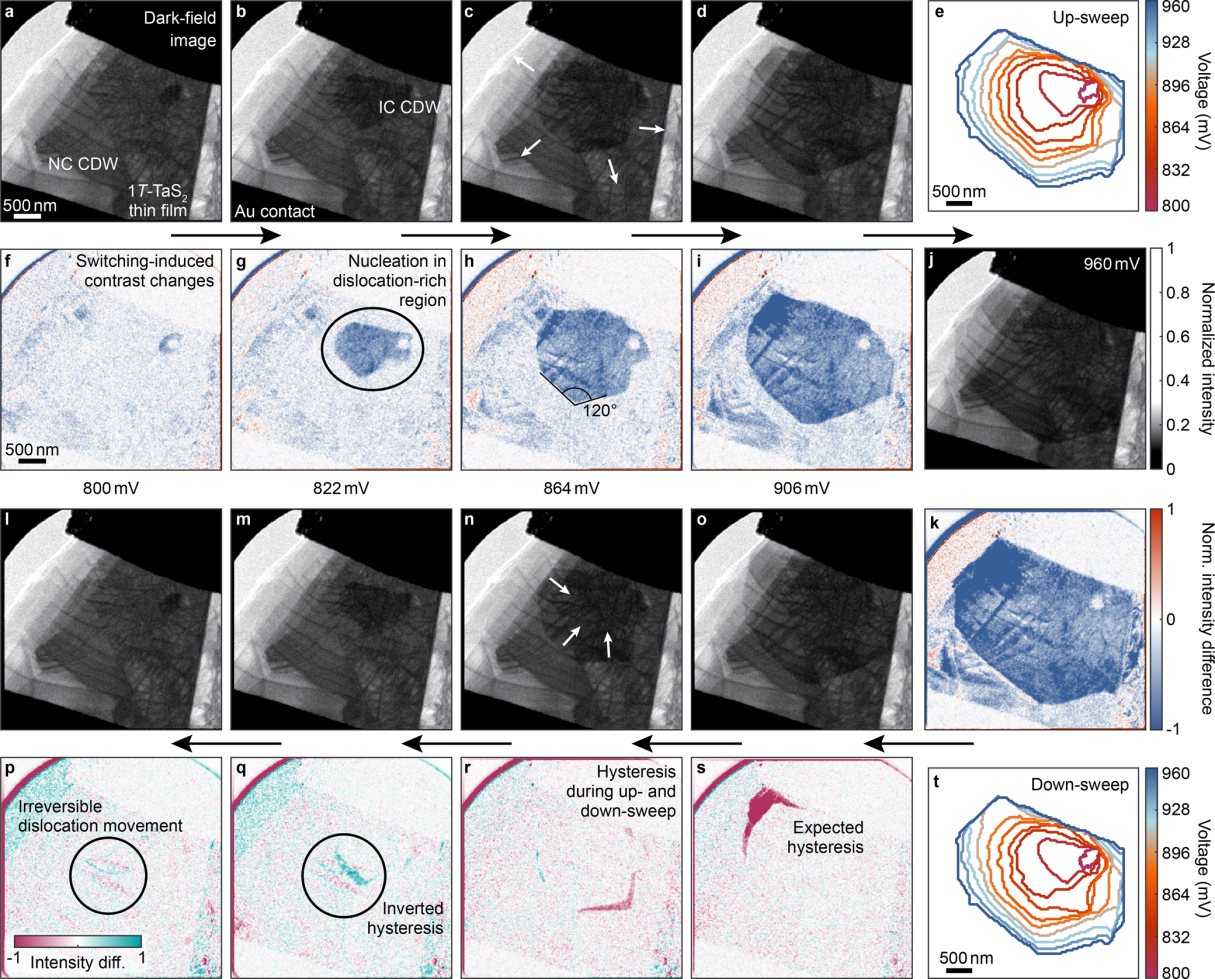
Current-induced charge-density wave phase switching. **a–d** Dark-field images during the voltage up-sweep. The two CDW phases can be distinguished by bright and dark contrast. **e** Extracted growth of the IC domain profile during the voltage increase. **f–i** Image contrast change during the current-induced switching, obtained by subtracting a reference image recorded with a bias below 760 mV. The IC domain profile (blue contrast) emerges first in the region with the highest dislocation density. **j,k** Maximum expansion of the IC domain at a bias of 960 mV in this measurement series. **l–o** Dark-field micrographs of the bias down-sweep. **p–s** Intensity difference of the images in **l**–**o** compared to the images in **a**–**d**, respectively. The hysteresis expected for a first-order phase transition is strongest in a homogeneous sample region (purple contrast), but can also be inverted (green contrast). The temperature sweeping of the device further induces dislocation movements (see the image in **p**). **t** Extracted evolution of the IC phase domain during the down-sweep.

Different mechanisms have been invoked to describe the electrically induced CDW transformations in 1*T*-TaS_2_,^40−42,48^ with strong evidence that Joule heating is the dominant process under DC bias.^53−55,91^ In our measurements, the sample temperature increase depends on a dynamical equilibrium of nanoscale heat generation and micrometer thermal dissipation into the gold contacts as well as the supporting silicon nitride membrane. For the measured average film thickness of 90 nm, we do not expect pronounced variations of transition temperatures and hysteresis width as a function of thickness as were observed previously for thinner samples.^31,41,42^ The IC phase domain then nucleates close to the upper contact (dark contrast in Figure 2a–d), which simultaneously represents a region of enhanced dislocation density. The subsequent growth upon increasing the voltage alternates between strong pinning and sudden jumps of the interphase boundary. The phase front remains fixed for a few voltage steps, followed by switching a larger fraction of the thin film by the next increase. In contrast, the dynamics at higher bias unfold more smoothly (Figure 2e and t), traversing a more homogeneous sample area with a larger dislocation separation. In contrast to the role of dislocations, we have not found evidence for stacking faults (in either the CDW or the host lattice) influencing the domain nucleation and growth for our device geometry. The corresponding phase switching along the depth of the 1*T*-TaS_2_ thin film is encoded in the image intensity. In our measurements, we only observe full contrast changes, indicating an abrupt transformation in the out-of-plane direction, in agreement with the mostly lateral temperature gradients.

Throughout the entire domain growth, there is no preference of the phase boundary to align with individual dislocations, in contrast to the previously observed pinning of the NC phase upon further cooling to the low-temperature commensurate PLD.^54^ The likely origin for this difference is that, in the latter case, both phases exhibit the same local texture, which may allow for dislocations decorated by NC-discommensurations.^83^ The propagating phase front in our measurements is unaffected by this form of structural heterogeneity. Instead, we observe characteristic 120° angles in the phase boundary (Figure 2h), similar to previous findings in laser-induced heating of a 1*T*-TaS_2_ thin film.^82^ It seems natural to associate these orientational preferences with the native behavior of the transformation,^68^ linking them to either the trigonal host lattice structure or the 6-fold symmetry of the CDW itself. Indeed, a correlation with the lattice structure available in electron diffractograms revealed an angular proximity to a glide plane in the 1*T*-TaS_2_ host lattice,^82^ warranting further investigations of the switching mechanism on atomic length scales. In light of this influence of the host lattice on the domain growth, such measurements could also clarify whether point-defects like impurity atoms, interstitials, and vacancies may interact strongly with the interphase boundary.

During the voltage up-cycle, we further observe irreversible dislocation movements (Figure 2p). These changes, however, occur at temperatures beyond the local phase transition threshold and only after the structural transformation in the respective sample regions. As a consequence, we attribute them to temperature-driven strain release.^55^

The domain evolution is mirrored in the device’s residual resistance, where a macroscopic signature of the first-order phase transition is not visible (Figure 3g, we subtracted a linear metallic contribution for better visibility). Instead, the switching induces a succession of smaller hysteretic features, coinciding with the evolution of the size and shape of the IC domain (Figure 3f), and a hysteresis inversion at around 860 mV.

**Figure 3:**
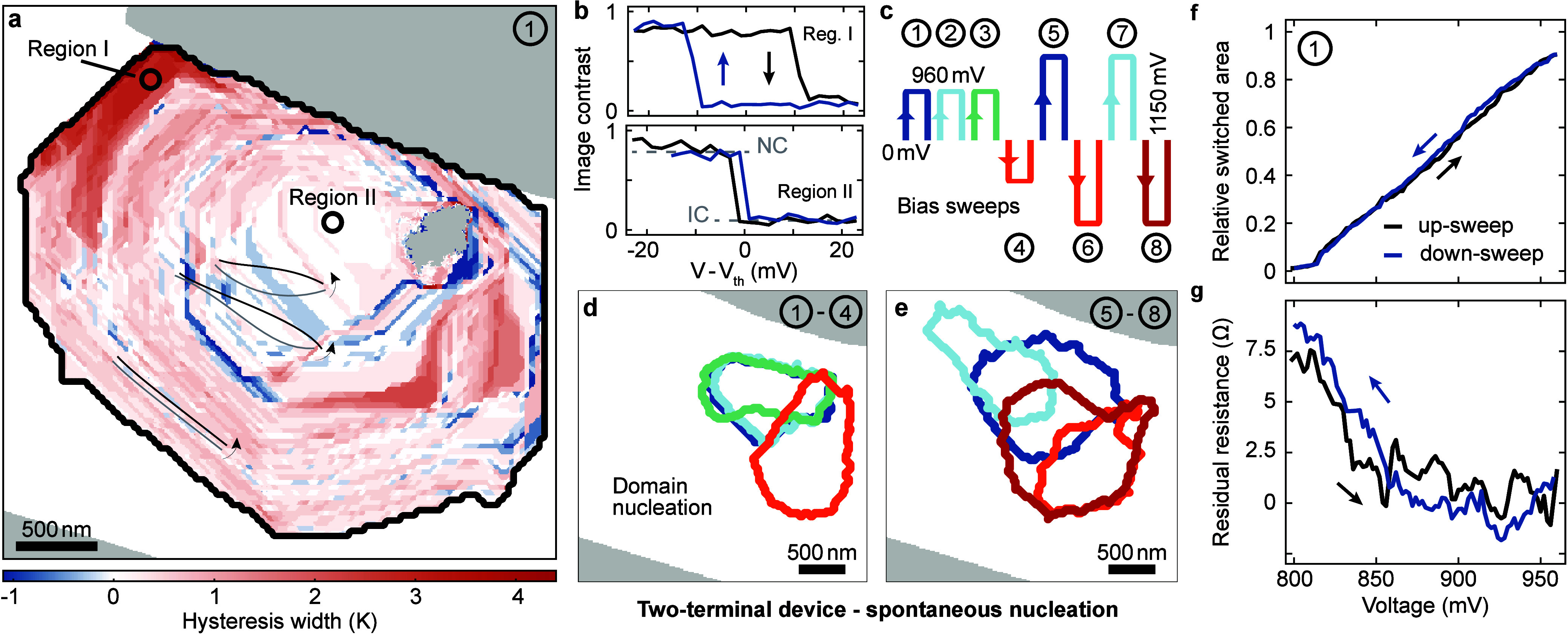
Hysteresis width mapping and domain nucleation in the two-terminal device. **a** Temperature hysteresis extracted from the evolution of the domain pattern, based on an ohmic behavior of the material. The nucleation region is characterized by local suppression of the phase transition threshold (white contrast). Gray regions indicate the gold contacts and the initial domain size at the lowest voltage applied. The black contour line represents the largest extent of the IC domain for this measurement. The bias sweep further induces dislocation slips (from gray to black lines, highlighted by the arrows). **b** Local voltage hysteresis width for the two regions highlighted in **a**, centered by the respective threshold voltage *V*_th_. **c** Bias sweep sequence applied to the device. Following three similar voltage ramps from 0 mV to 960 mV, we repeatedly reverse the polarity and increase the maximum bias to 1150 mV. **d,e** Domain pattern nucleation. The colored lines correspond to the outline of the first IC domain of considerable size during the bias up-sweeps. While the subsequent domain growth evolves similarly during the first sweeps (measurements 1–3), a polarity inversion and a larger maximum bias change the phase formation at every cycle (4–8). **f** Domain size extracted from the DF measurements. **g** Macroscopic electrical characterization. The residual resistivity is obtained by subtracting a linear metallic contribution from the measured resistances. As the local phase switching is governed by the microstructure, we find a sequence of hysteretic features rather than a single hysteresis loop.

The direct observation of the phase nucleation and the dense sampling of the subsequent domain growth also provide a quantitative measure of the energy scales involved. Specifically, reconstructing the temperature profiles across the flake during the voltage up- and down-sweeps, we map local variations of the hysteresis width Δ*T*(*r⃗*) of the weakly first-order transition. To this end, we consider an ohmic behavior of the material and calculate the local threshold voltage based on the IC phase domain evolution (see Supporting Information for a detailed description). The result is depicted in Figure 3a. Overall, we find pronounced variations over the micrometer flake. In the outer regions of a large domain, we observe the hysteresis expected for a first-order phase transformation (red contrast), albeit that the expected width of 4 K^29^ is only reached when strong pinning precedes a subsequent pronounced IC domain growth (see also Figure 3b). Similarly, the described irreversible dynamics also affect the transformations between the two CDW phases. The regions with the most prominent dislocation slips display a seemingly inverted hysteresis, possibly arising from locally changed strain and the resulting critical temperature (blue contrast and line overlays in Figure 3a; see also Figure 2p and q). The central part of the flake, on the other hand, is characterized by a hysteresis suppression or even its absence (white contrast), as the strain profile induced by the enhanced defect density locally reduces the phase transition barrier.^6^

The distinct microstructure of the material thus favors a spontaneous nucleation of the high-temperature IC phase domain. Furthermore, the seeding is unstable against small changes in the external electrical drive. Figure 3d and e display the outline of the IC domain after the nucleation for a total of eight different bias sweeps (see also Videos 3 to 10 and Figure 3c). During the first three of these cycles, similar voltage ramps bring about an almost identical domain evolution (blue and green outlines in Figure 3d). A polarity inversion combined with a higher maximum bias, however, varies the direction of the initial domain growth (orange outline in Figure 3d and Figure 3e), thus deviating from a purely ohmic behavior (and therefore Joule heating)^53−55,91^ of the phase transformation. Our measurement scheme is particularly sensitive to such local variations that may originate from a combination of different influences on the phase transformation mechanism. Specifically, the phase profiles observed for a given polarity are not completely identical (blue and green outlines vs red color tones). Nevertheless, there is a larger similarity between domains recorded under the same current flow direction, compatible with an electrical-field dependence of the phase nucleation, possibly enabled by the locally reduced transition barrier across a considerable part of the flake. All in all, these results demonstrate that the alterations to the free-energy landscape of, in particular, second- and weakly first-order structural transformations imposed by nanoscale heterogeneity compromise the repeated reversible switching of structural transformations.

In contrast, for most technological applications, a deterministic, abrupt, and hysteretic phase change may be more desirable. As we show in a second set of measurements, such a behavior can be enforced by a different device geometry. The investigated sample has a similar thickness (Figure S3), but shows considerably less strain and a smaller contact gap (Figure 4a), and the two gold leads are electrically connected via a thin gold wire. Since the current preferentially flows through the metallic short, the wire acts as a local heater during the experiments such that the phase nucleation occurs in close proximity to the corresponding sample edge. Supported by the enhanced directionality of the current flow, we observe the described characteristic angles in the interphase boundary throughout the entire domain growth (Figure 4e–g). Also the macroscopic behavior of the shorted device is much closer to the case suggested by bulk properties,^92^ as we find a pronounced hysteresis in both the average image intensity (derived from the data set displayed in Supplementary Video 11; see also Figure 4b) and the residual resistance (Figure 4c and d).

**Figure 4:**
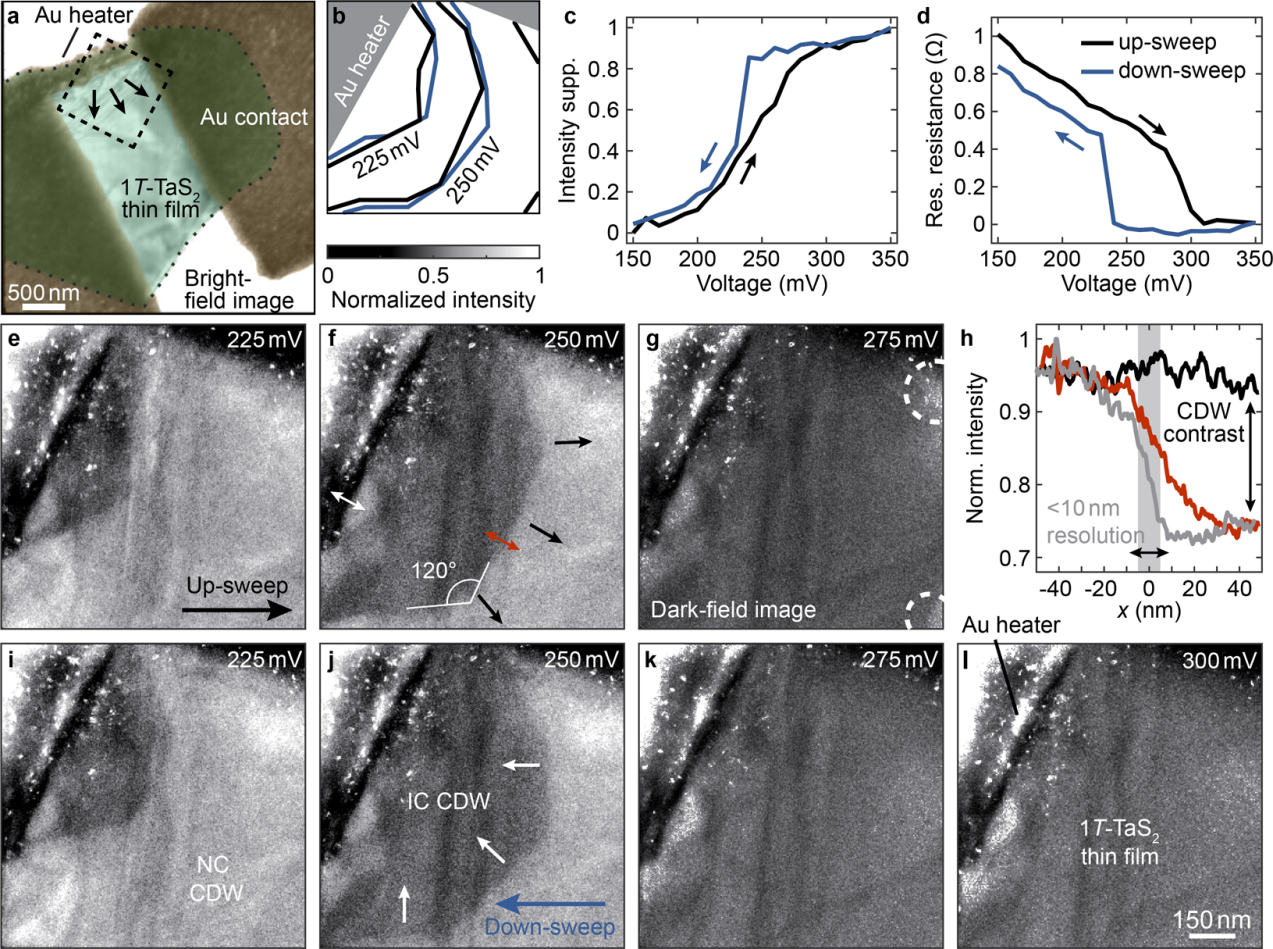
Controlled IC phase nucleation in a shorted device geometry. **a** Electron micrograph of the shorted device. A thin gold wire connected the two contacts. Note the enhanced overall homogeneity of the 1*T*-TaS_2_ thin film compared to the two-terminal device. **b** Interphase boundary extracted from the DF images, highlighting the hysteresis in the switched area between the up- and the down-sweep. **c** Relative image intensity suppression during the bias sweep. **d** Macroscopic electrical characterization, showing a pronounced hysteresis in the residual resistance where the metallic contribution of the gold wire is subtracted from the recorded curve. **e–g** Dark-field micrographs of the bias up-sweep and within the sample region highlighted by the dashed box in **a**. Since the current largely flows through the gold wire, the short acts as a heater, resulting in preferential nucleation of the IC phase close to the corresponding sample edge. **h** Intensity line-out across the NC-to-IC interphase boundary (red) highlighted in **f** compared to an intensity profile in the same region before the phase transformation (black). For reference, the gray curve displays a sharper line-out across the gold heater (white arrow in **f**), indicating that the phase boundary is spatially resolved. **i–l** DF images recorded during the voltage down-sweep.

In conclusion, we present combined TEM and electrical transport measurements in two different 1*T*-TaS_2_ devices. Our results outline routes for the controlled switching of structural transitions, emphasizing the impact of device geometry and, particularly, nanoscale heterogeneity. As demonstrated here, tailored DF imaging is a powerful tool to visualize electrically induced phase switching and to link it to the underlying microstructure.

Beyond investigations of electrically induced phase transformations, the customized DF aperture holder further opens up possibilities to investigate the effects of different external stimuli, or a combination thereof, with nanometer spatial resolution. First experiments employing simultaneous optical and electrical excitation of strongly correlated materials, the backbone of optoelectronic devices,^57−59^ suggest an intricate response of the structural degrees of freedom to such schemes.^93^ An alternative method to access these processes is given by four-dimensional scanning transmission electron microscopy (“4D-STEM”),^94,95^ rastering an electron nanobeam over the sample while recording a diffractogram at every scan position. Such measurements routinely enable the correlation of various structural features^72,96,97^ and the extraction of electric fields on the atomic scale.^98^ We consider tailored DF imaging a complementary technique, as it allows for a real-time and full-field observation of structural changes. The ability to correct for sample drifts between individual acquisitions favors imaging with low-intensity beams that require longer exposure times.^82^ As a result, tailored DF imaging is particularly suited for tracing structural dynamics on ultrafast^82,99−105^ or nanosecond time scales.^21,55,106^

More generally, our approach expands the possibilities to directly image nanoscale processes that are encoded in complex structural signatures in *operando* or *in situ* investigations of spatially heterogeneous systems. Finally, electron-optical elements beyond DF filters will benefit from enhanced alignment flexibility, including phase plates designed to enhance resolution and contrast in electron microscopes.^107−110^

## Supporting Information Available

Fabrication and characterizations of the electrically contacted samples; details on the local heater geometry in the second device; details on the transmission electron microscopy measurements; fabrication of dark-field apertures; extraction of the nanoscale thermal hysteresis; evolution of electron diffractograms during the voltage sweeps (PDF)
Supplementary Video 1 (MP4)
Supplementary Video 2 (MP4)
Supplementary Video 3 (MP4)
Supplementary Video 4 (MP4)
Supplementary Video 5 (MP4)
Supplementary Video 6 (MP4)
Supplementary Video 7 (MP4)
Supplementary Video 8 (MP4)
Supplementary Video 9 (MP4)
Supplementary Video 10 (MP4)
Supplementary Video 11 (MP4)
