# Supplementary material for: Nanoscale Operando Imaging of Electrically Driven Charge-Density Wave Phase Transitions
Source: Nano Lett. 2024 Sep 24;24(40):12476–85. doi: 10.1021/acs.nanolett.4c03324 (PMC11468880; doi:10.1021/acs.nanolett.4c03324)
Supplement: Supplementary file 1 — nl4c03324_si_001.pdf [file nl4c03324_si_001.pdf]

Supporting Information for

Nanoscale *Operando* Imaging of Electrically Driven Charge-Density Wave Phase Transitions

Till Domröse,[†,‡] Noelia Fernandez,[¶,@] Christian Eckel,[¶,@] Kai Rossnagel,[§,‖] R. Thomas Weitz,[¶,⊥] and Claus Ropers[*,†,#]

†*Department of Ultrafast Dynamics, Max Planck Institute for Multidisciplinary Sciences, 37077 Göttingen, Germany*

‡*4th Physical Institute — Solids and Nanostructures, University of Göttingen, 37077 Göttingen, Germany*

¶*1st Institute of Physics, University of Göttingen, 37077 Göttingen, Germany*

§*Institute of Experimental and Applied Physics, Kiel University, 24098 Kiel, Germany*

‖*Ruprecht Haensel Laboratory, Deutsches Elektronen-Synchrotron DESY, 22607 Hamburg, Germany*

⊥*International Center for Advanced Studies of Energy Conversion (ICASEC), University of Göttingen, 37077 Göttingen, Germany*

#*4th Physical Institute – Solids and Nanostructures, University of Göttingen, 37077 Göttingen, Germany*

@*N.F. and C.E. contributed equally to this work.*

E-mail: claus.ropers@mpinat.mpg.de

Fabrication of electrically-contacted samples

$1T$-TaS_2 thin films were prepared by mechanical exfoliation of bulk crystals using blue SWT20+ "Nitto" tape. The $1T$-TaS_2 crystals used for the samples discussed in the main text (also Figure S1c,d) were provided by Kai Rossnagel's group at Kiel University. As TEM sample supports, we use commercially available Si_3N_4 substrates ("Norcada") with a thickness of 20 nm. In order to characterize the electrically-induced CDW phase transformations, we prepared samples such as the ones displayed in Figure S1. $1T$-TaS_2 thin films are placed in the center of the electron-transparent window of the TEM support, and electrically contacted with two-terminal leads (4 nm Ti/ 90 nm Au) via electron beam lithography and physical vapor deposition. The presence of CDW order is subsequently verified in TEM diffraction measurements, recognizable by the characteristic, low-intensity satellite spots (green circles in Figure S1b).[1] In particular, Figure S1c and d display the two-terminal device discussed in the main text that is displayed in Figures 1-3, recorded by atomic-force and optical microscopy, respectively. The $1T$-TaS_2 film has a thickness of around 90 nm. For the shorted device displayed in Figure 4, we determine the thickness of the $1T$-TaS_2 thin film by TEM measurements. Specifically, we record two images with a camera attached behind an energy filter. The first image is unfiltered, i.e., both elastic and inelastic electron scattering contribute to the image contrast. For the sec-

ond image, we filter out the inelastic part of the spectrum. The contrast ratio of the two images allows for a spatially-resolved determination of the sample thickness as the inelastic scattering in the material is linked to the electron mean-free path length (t/λ measurement).[2] For a convergence angle of 0.1 mrad and a collection angle of 15 mrad, we calculate mean-free path lengths of 96 nm and 116 nm for $1T$-TaS_2 and Si_3N_4, respectively. The estimated sample thickness based on these measurements is displayed in Figure S3. Assuming that the Si_3N_4-membrane underneath has a fixed thickness of 20 nm, we find an average thickness for the $1T$-TaS_2 thin film of 95 nm.

In addition to the electrical characterizations alongside the TEM imaging described in the main text, CDW phase switching in these devices is investigated by temperature sweeps in a closed-cycle Lakeshore probe system with a temperature scanning range between 5 K and 400 K. The structural transition in a third device between the material's NC phase and the low-temperature commensurate (C) CDW results in a pronounced increase in the electrical resistance (Figure S1a).[3] The resistance is derived by recording current-voltage curves up to a maximum bias of 1.5 mV to minimize additional Joule heating.

Local heater geometry

Figure S2 shows the electrical characterization of the second device discussed in the main text. The measured differential resistances are on the order of 100 Ω. In the DF measurements, we observe an IC phase nucleation close to the metallic short above a critical voltage, followed by an almost instantaneous switching within the entire field-of-view for the next few voltage increases. Therefore, we conclude that a large fraction of the current flows through the metallic short, leading to preferential Joule heating in the corresponding sample edge. The $1T$-TaS_2 thin film, however, also contributes to the electrical properties, as the flake temperature is already close to the critical value when the phase nucleation sets in. Consequently, a small additional voltage change suffices to induce the phase transformation globally.

This characteristics is mirrored in the measured resistance displayed in Figure S2. The metallic contribution of the gold wire is evident from the linear resistance change with increasing voltage, while the instantaneous phase switch brings about the well-defined hysteresis loop (Figure S2b). In the main text, we discuss the residual resistance for both devices, i.e., the linear, metallic contribution is subtracted from the displayed curves. To this end, we fit a linear function to the measured resistances at larger voltages above the critical value, resulting in the curve displayed in Figure S2c. For comparison, the electrical characterization of the two-terminal device discussed in the main text is displayed in Figure S4e.

Transmission electron microscopy measurements

For the fabrication of the tailored dark-field aperture arrays, we used commercially available TEM silicon nitride support films with a window-size of 500 µm × 500 µm and a membrane thickness of 50 nm. Depositing a gold layer with a nominal thickness of 650 nm by argon sputtering ensured that the sample carriers were not transparent for electrons accellerated to 120 keV. The size of the aperture array was calibrated based on an electron diffractogram of the NC phase, and etched into the gold film by focused ion beam milling. Accounting for both types of the mirror-symmetric CDW orientation of the NC phase in $1T$-TaS_2,[1] we positioned both an array tailored to the α as well as to the β configuration in the same gold film (Figure S4).

Using the custom DF aperture holder, the masks are positioned in the back-focal plane of the objective lens and aligned to the diffractograms obtained from the specimen in the microscope's diffraction mode (Figure S4b). As described in the main text, the analogue DF filter provides selective contrast to the local NC CDW amplitude by means of an array of 72 individual apertures whose distribution corre-

sponds to the position of the brightest NC reflections in the diffractograms. As the projected diameter of the individual apertures is smaller than 0.04/Å, electrons scattered into second-order IC or the main lattice reflections are blocked. The alignment of the filter to the electron diffractograms available in the back-focal plane of the objective lens includes three translational and the in-plane rotational degree-of-freedom provided by the custom-made aperture holder. Simultaneously, trimming the beam in reciprocal space defines the spatial resolution in the images.[4]

Combined TEM and electrical transport measurements were conducted in a "JEOL JEM-2100F" equipped with a ZrO/W Schottky emitter, operated at a beam energy of 120 keV. The fabricated samples were transferred into a custom-made TEM sample holder that allows electrical biasing. An electrical connection to the sample was established with an ultrasonic "K&S 4523 Wedge Bonder". The dark- and bright-field micrographs of the first electrical device, displayed in Figures 1-3, were recorded with a direct electron detector ("Direct Electron DE-16") and processed via an electron counting algorithm. For the TEM images of the second device (Figure 4) we used a "TVIPS XF416" attached behind an energy filter for removing the inelastically scattered electrons from the images ("CEOS CEFID").

In addition to the DF imaging described in the main text, we evaluate the current-induced CDW phase switching based on electron diffractograms recorded at different bias (Figure S5). The phase transformation is recognizable in the suppression of the second-order PLD satellite reflections, including the structural hysteresis that is also found in the electrical characterization for the shorted device.

Extracting the nanoscale hysteresis

Based on the extraction of the IC phase domain during the densely sampled voltage upan down-sweep, we extract the temperature profiles at the highest voltage applied, and map them onto nanoscale variations of the phase transition hysteresis. In these measurements, and starting from room-temperature T_0, the structural phase transformation is induced when the local temperature $T(\vec{r})$ exceeds the critical temperature T_{th}. In particular, this applies to the outermost regions of the largest detected domain under the maximum bias V_{max}. For every voltage change ΔV, we then identify the difference between the domain pattern at the voltages V and $V-\Delta V$, and upscale the temperature in these regions by

$$g_{\mathrm{up,down}}(\vec{r}) = (T_{\mathrm{th}} - T_0)\left(\frac{V_{\mathrm{max}}}{V}\right)^2 . \tag{S1}$$

This approach models an ohmic behaviour of the contacted $1T$-TaS_2 flake, without the relatively small resistance change associated with the structural transformation.[5] We obtain the profiles displayed in Figure S6 that correspond to the temperature distributions at V_{max}, and, importantly, yield spatially dependent temperature scalings $g_{\mathrm{up,down}}$ for voltages $V < V_{\mathrm{max}}$. In particular, these maps allow to define the local threshold voltage V_{th} associated with a temperature increase

$$T_{\mathrm{th}} - T_0 = g(\vec{r})\left(\frac{V_{\mathrm{th}}(\vec{r})}{V_{\mathrm{max}}}\right)^2 , \tag{S2}$$

where $g(\vec{r}) = \frac{1}{2}(g_{\mathrm{up}} + g_{\mathrm{down}})$. The hysteresis width $\Delta T(\vec{r})$ is then obtained by considering the difference between the local threshold temperatures during the up- and down-sweep, reached at the local threshold voltage, i.e.,

$$T_{\mathrm{th,(up,down)}} = g_{\mathrm{up,down}}(\vec{r})\left(\frac{V_{\mathrm{th}}(\vec{r})}{V_{\mathrm{max}}}\right)^2 . \tag{S3}$$

Consequently,

$$\Delta T(\vec{r}) = T_{\mathrm{th,down}}(\vec{r}) - T_{\mathrm{th,up}}(\vec{r}) \tag{S4}$$

$$= \left[g_{\mathrm{down}}(\vec{r}) - g_{\mathrm{up}}(\vec{r})\right]\frac{T_{\mathrm{th}} - T_0}{g(\vec{r})} . \tag{S5}$$

The result is depicted in Figure 3a in the main text.

Supplementary Videos

Supplementary Videos 1 and 2 Charge-density wave nanoimaging and difference images for the voltage up- and down-sweep discussed in the main text and Figure 2.

Supplementary Videos 3–10 Dark-field microscopy videos and difference images for data sets 2–8 as specified in Figure 3. Videos 9 and 10 contain the sweep sequences 5–8.

Supplementary Video 11 Characterization of the second device displayed in Figure 4.

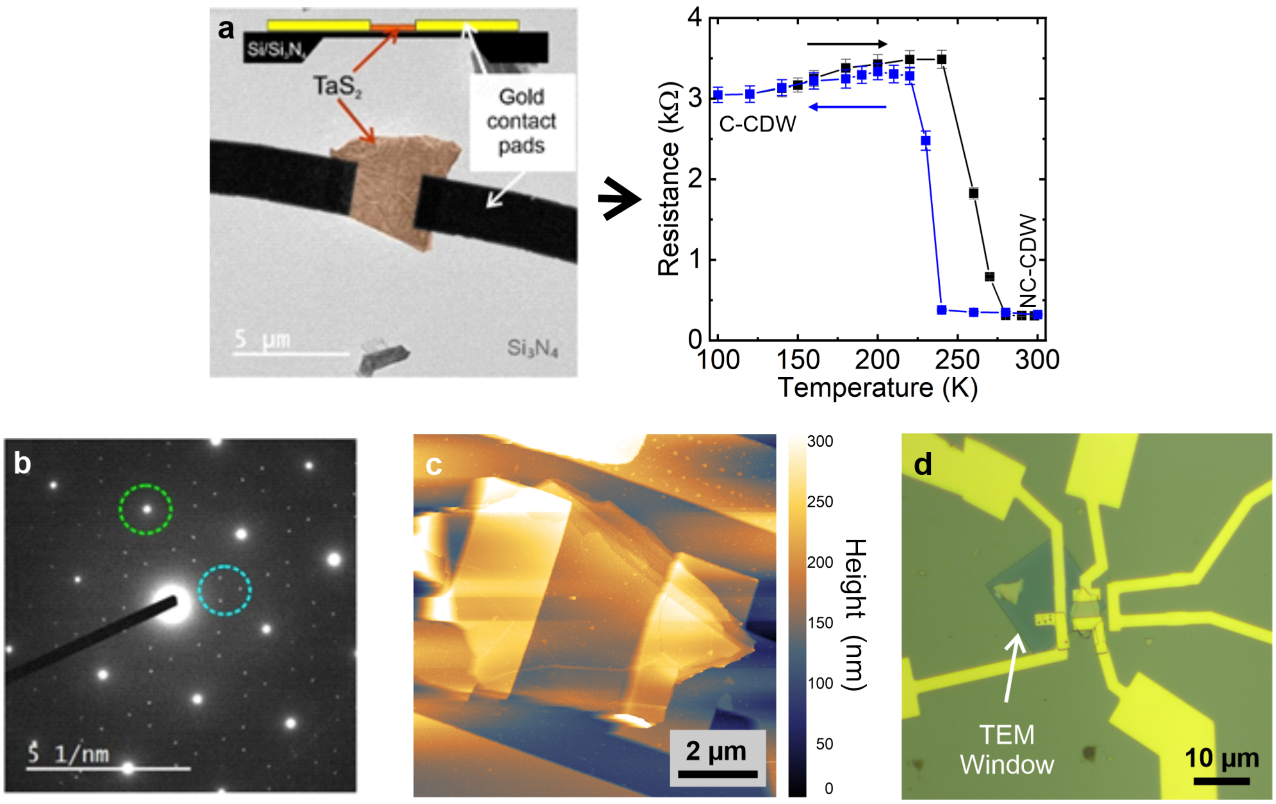

Figure S1: **Sample fabrication and characterization.** **a** Bright-field TEM image of the device and a side-view scheme. The flake with gold contacts sits on the TEM window (Si_3N_4 membrane). The presence of CDW phases is confirmed by a temperature dependent transport measurement (right side). **b** Diffraction image of the flake in **a**. The characteristic low-intensity NC-CDW satellite spots are observed at room temperature (cyan dashed circle) around the brighter main lattice reflections (green dashed circle). **c** AFM measurement of the flake in **d** with a thickness of $\sim 90\,\mathrm{nm}$. **d** Optical microscope image of the two-terminal $1T$-TaS_2 device discussed in the main text.

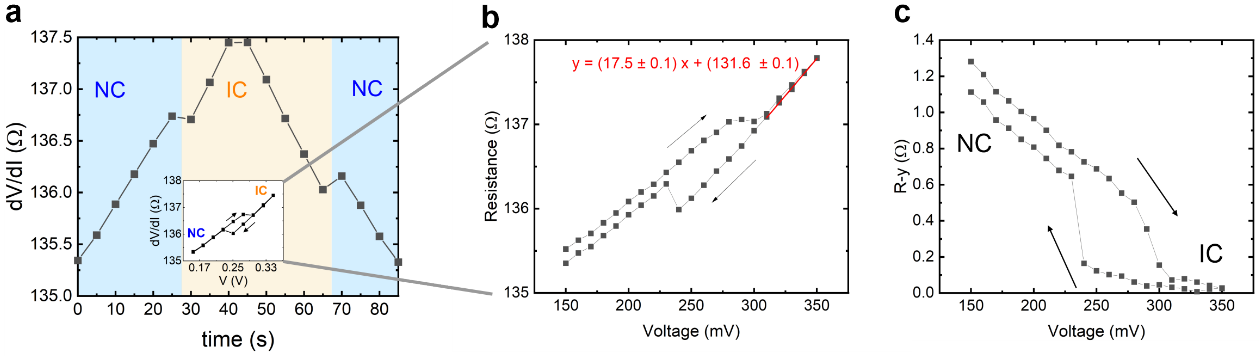

Figure S2: **Electrical raw data of local heater geometry.** **a** Resistance of the sample measured at time intervals of 10 s. The inset shows the datapoints as a function of the voltage applied between the two contacts. For each datapoint, a dark-field TEM image was taken, as discussed in the main text. **b** Linear fit (solid red line) of the metallic part of the device. **c** Residual resistance obtained after subtracting the metallic contribution from the measured resistance.

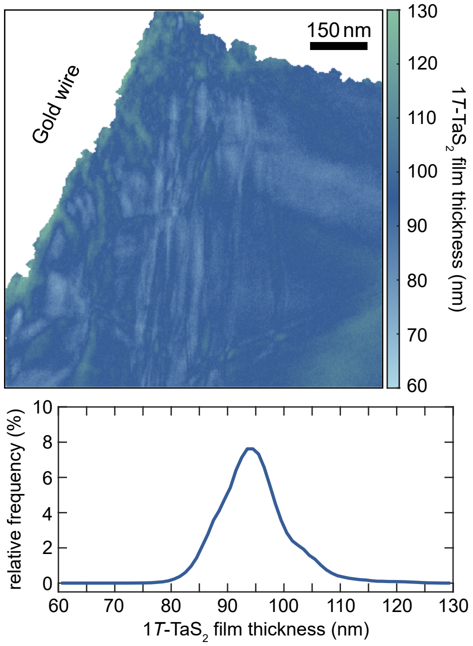

Figure S3: Thickness determination of the shorted device discussed in the main text (see also Figure 4). The average thickness of the $1T$-TaS_2 film amounts to 95 nm. For the derivation of the histogram (bottom), we choose a bin size of 1 nm.

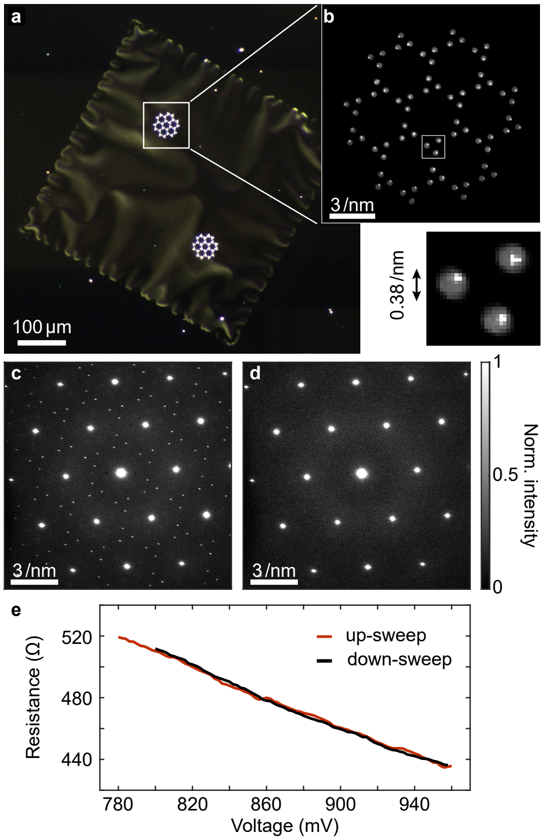

Figure S4: **Dark-field masks.** **a** Image of the DF aperture arrays built into the custom aperture holder. The prepared TEM sample carrier hosts two masks, accounting for both mirror-symmetric orientations of the NC phase in 1 T-TaS_2. **b** Masked electron diffractogram of the NC CDW phase as accessible in the back-focal plane of the objective lens. The projected aperture diameter amounts to below 0.04/Å. **c** Electron diffractogram of the NC CDW phase of 1 T-TaS_2. **d** Electron diffractogram of the IC CDW phase. **e** Resistance measurement for the two-terminal device discussed in the main text.

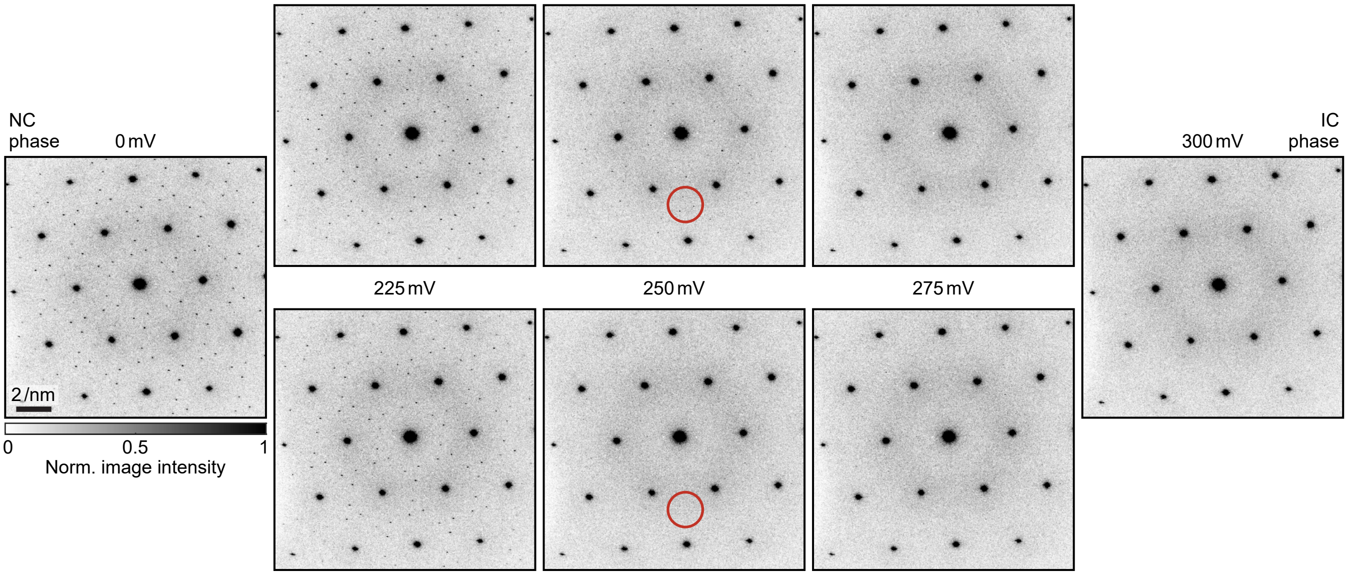

Figure S5: Electron diffractograms of $1T$-TaS_2 at different voltages applied to the shorted device discussed in the main text. The top row corresponds to the voltage up-sweep, and the bottom row to the down-sweep. Note the absence of CDW satellite spots in the down-sweep at 250 mV, illustrating the hysteresis of the phase transformation.

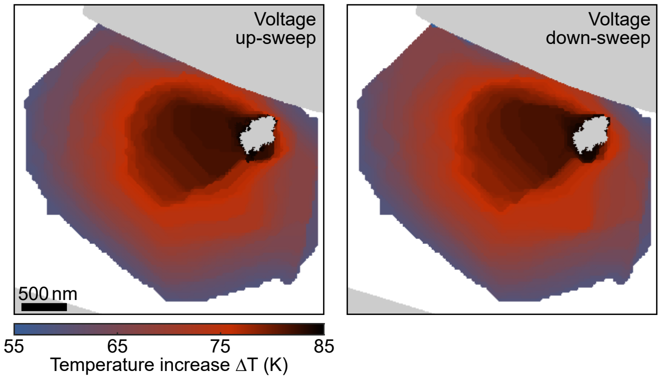

Figure S6: Temperature increase at the highest voltage applied, extracted from the bias up- (left) and down-sweep (right) described in the main text.

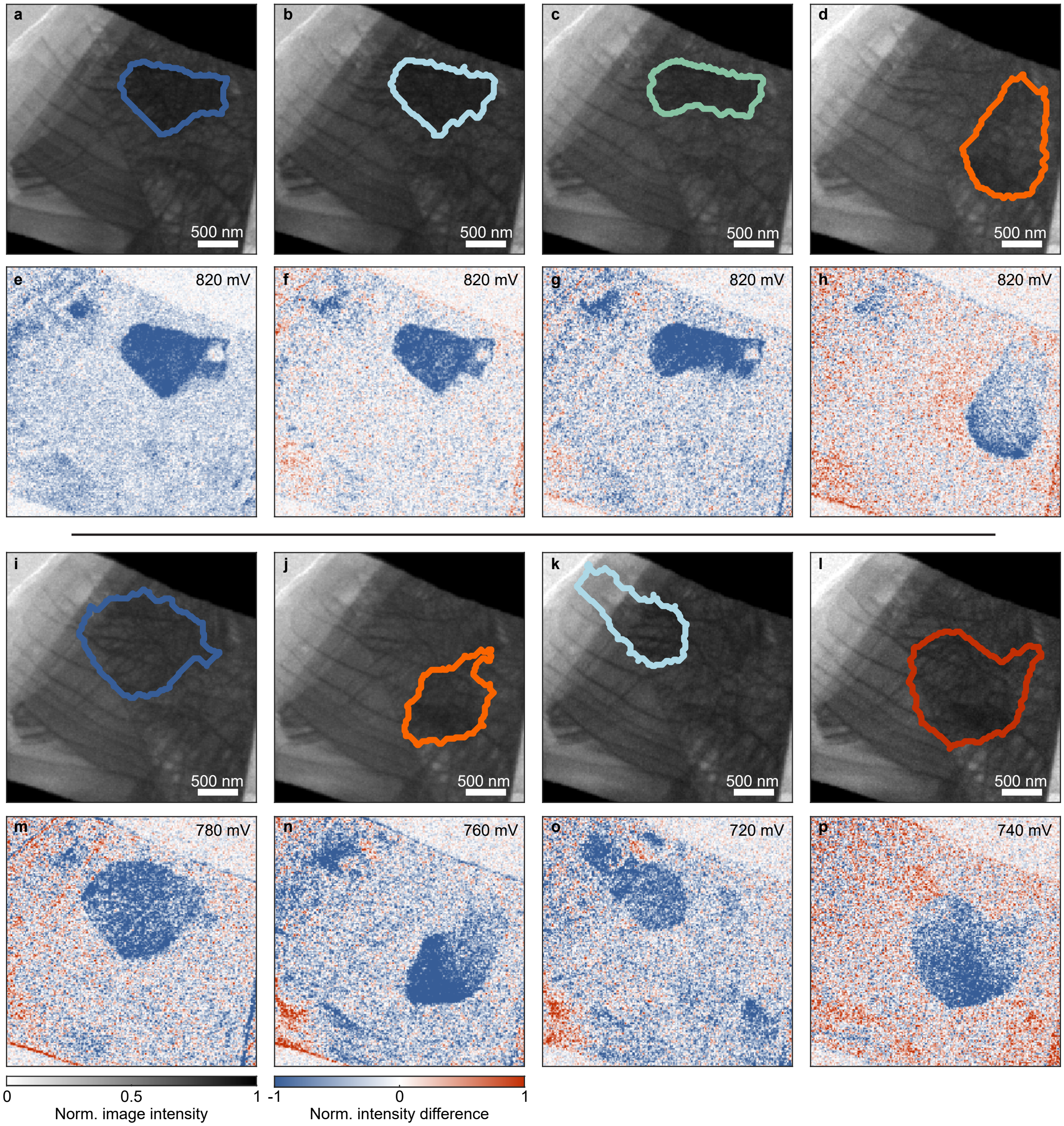

Figure S7: **Field-dependence of CDW domain seeding in 1T-TaS_2 a-d** and **i-l** Dark-field micrographs and domain lineouts corresponding to the measurements displayed in Figure 3**d,e** in the main text. **e-h** and **m-p** Intensity difference images obtained by subtracting a reference image recorded for a bias of 0 mV.

References

(1) Spijkerman, A.; de Boer, J. L.; Meetsma, A.; Wiegers, G. A.; van Smaalen, S. X-Ray Crystal-Structure Refinement of the Nearly Commensurate Phase of $1T$-TaS_2 in (3+2)-Dimensional Superspace. *Physical Review B* **1997**, *56*, 13757–13767.

(2) Egerton, R. *Electron Energy-Loss Spectroscopy in the Electron Microscope*; Springer US: Boston, MA, 2011.

(3) Sipos, B.; Kusmartseva, A. F.; Akrap, A.; Berger, H.; Forró, L.; Tutiš, E. From Mott State to Superconductivity in $1T$-TaS_2. *Nature Materials* **2008**, *7*, 960–965.

(4) Danz, T.; Domröse, T.; Ropers, C. Ultrafast Nanoimaging of the Order Parameter in a Structural Phase Transition. *Science* **2021**, *371*, 371–374.

(5) Hellmann, S.; Beye, M.; Sohrt, C.; Rohwer, T.; Sorgenfrei, F.; Redlin, H.; Kalläne, M.; Marczynski-Bühlow, M.; Hennies, F.; Bauer, M.; Föhlisch, A.; Kipp, L.; Wurth, W.; Rossnagel, K. Ultrafast Melting of a Charge-Density Wave in the Mott Insulator $1T$-TaS_2. *Physical Review Letters* **2010**, *105*, 187401.
